# Detection, quantitation, and genotyping of human papillomavirus circulating tumor DNA by droplet digital PCR

**DOI:** 10.1128/jcm.00585-25

**Published:** 2025-08-19

**Authors:** Emily C. Fernholz, David M. Routman, Kathryn M. Van Abel, Eric J. Moore, Daniel J. Ma, Danielle E. Hunter, Kathleen R. Bartemes, James S. Lewis, Erik B. Wendlandt, Matthew J. Binnicker

**Affiliations:** 1 Division of Clinical Microbiology, Department of Laboratory Medicine and Pathology, Mayo Clinic 195112 , Rochester, Minnesota, USA; 2 Department of Radiation Oncology, Mayo Clinic 189709 , Rochester, Minnesota, USA; 3 Department of Otolaryngology-Head and Neck Surgery, Mayo Clinic 314210 , Rochester, Minnesota, USA; 4 Division of Anatomic Pathology, Department of Laboratory Medicine and Pathology, Mayo Clinic Arizona 23387, Scottsdale, Arizona, USA; 5 Integrated DNA Technologies 10865 Coralville, Iowa, USA; St Jude Children's Research Hospital, Memphis, Tennessee, USA

**Keywords:** molecular, genotyping, screening, head and neck cancer, HPV

## Abstract

Human papillomavirus (HPV) is comprised of >200 genotypes and has an ~8 kb, circular, double-stranded DNA genome. Transmission of HPV occurs through skin-to-skin contact and infection of squamous epithelial cells of cutaneous and mucosal surfaces. HPV genotypes are categorized as low- or high-risk (hrHPV) based on oncogenic potential. There are approximately 14 types of hrHPV that can cause several types of cancer, including HPV-associated oropharyngeal squamous cell carcinoma (HPV(+)OPSCC). Detection of HPV(+)OPSCC is traditionally accomplished using p16 immunohistochemistry (IHC) and HPV-specific testing, either DNA or RNA *in situ* hybridization (ISH) staining or DNA-based PCR of suspected tumor biopsy tissue. More recently, platelet-poor plasma (PPP) samples from patients with HPV(+)OPSCC have proven useful for detection and quantitation of fragments of HPV circulating tumor DNA (ctDNA). ctDNA has been shown to be useful in determining treatment response and monitoring for disease recurrence. In this study, a novel droplet digital PCR assay (ddPCR) was developed and validated for the detection and quantitation of ctDNA from 5 hrHPV genotypes in PPP. Analytical sensitivity ranged from 7.71 to 19.45 fragments of HPV ctDNA per milliliter of PPP across five hrHPV genotypes. In patients with confirmed primary or recurrent HPV(+)OPSCC or HPV(-)OPSCC, testing of corresponding PPP samples (*n* = 32) by ddPCR demonstrated 90.63% (29/32) overall agreement with p16/HPV-ISH biopsy results. Compared with reference ddPCR assays performed at outside laboratories, our ddPCR assay yielded 90% (9/10) overall agreement. This assay may provide clinicians with a tool for monitoring HPV ctDNA prior to, during, and after treatment of an HPV-associated cancer.

**IMPORTANCE:**

At least 14 genotypes of human papillomavirus (HPV) have been identified to have high oncogenic potential. While molecular diagnostic testing for HPV is widely available for liquid cytologic cervical samples, testing is limited for other sample types, including liquid biopsy samples, such as platelet-poor plasma (PPP). With the rising incidence of HPV-associated oropharyngeal squamous cell carcinoma (HPV(+)OPSCC), laboratory testing is an essential part of patient diagnosis, management, and surveillance. Here, we summarize the development and analytical performance validation of a multiplexed, droplet digital PCR (ddPCR) assay for the detection and quantitation of HPV circulating tumor DNA (ctDNA) in PPP. This assay may provide clinicians with a tool to address minimal residual disease for patients with an HPV-associated cancer.

## INTRODUCTION

Human papillomavirus (HPV) may cause transient, asymptomatic infections, warts on dermal and mucosal surfaces, and various types of cancer. Approximately 42 million Americans between the ages of 15 and 59 years are infected with at least one disease-associated HPV genotype (1). While infection with HPV may be cleared by the immune system, some high-risk genotypes (e.g., HPV-16,-18, -31, -33, and -35, etc.) are able to repress cell modulator genes. Resulting tumorigenesis is most often associated with enhanced expression of viral E6 and E7 oncoproteins, as well as frequent integration of HPV into the host genome (2). Most nucleic acid-based tests cleared by the Food and Drug Administration for HPV detection target these oncogenes but are only cleared for cytologic cervical samples (3). HPV-associated cervical cancer has been well described, but other HPV-associated cancers are on the rise, including oropharyngeal squamous cell carcinoma (HPV[+]OPSCC), which has surpassed cervical cancer as the most common HPV-associated cancer in the United States (4). However, testing is not widely available for alternate sample types to assess primary infection or serial monitoring for an HPV-associated cancer. Diagnosis of an HPV-associated tumor, such as HPV(+)OPSCC, is often based on biopsy of the affected tissue, with treatment consisting of surgery, radiotherapy, and/or chemotherapy (5). Recently, detection of HPV circulating tumor DNA (ctDNA) has shown promise as a relatively non-invasive way to monitor treatment response and disease recurrence (6). Fragmented HPV ctDNA is released into circulation by apoptotic tumor cells and can be recovered in liquid biopsy samples, such as platelet-poor plasma (PPP).

In this study, we sought to develop and validate a droplet digital PCR (ddPCR) assay for the detection and quantitation of ctDNA from five high-risk HPV genotypes (HPV-16, -18, -31, -33, and -35) in PPP. A review of prior studies on HPV genotyping in head and neck carcinomas across different geographic regions showed that HPV-16 constitutes 88.4% of all HPV-associated cases, while a combination of HPV-18,-31, -33, and -35 is associated with an additional 7.2% cases. Therefore, these five high-risk types are likely to be associated with >95% of all head and neck carcinomas caused by HPV (7–14). The use of ddPCR allows for sensitive detection and precise quantitation compared to real-time PCR (i.e., qPCR). This method uses water-oil droplet emulsion technology to partition PCR reactions into thousands of droplets, which decreases the effect of potential inhibitors or contaminants and allows for amplification of low-abundance copies of DNA.

## MATERIALS AND METHODS

### Study design

Plasma samples (*n* = 32) were collected as part of a study reviewed and approved by the Institutional Review Board at Mayo Clinic (IRB# 22-000684; #19-006036). Clinical plasma samples were collected in EDTA (*n* = 29) or Biomatrica LBgard Blood Tube (*n* = 3) from patients who underwent tumor tissue biopsy testing using p16 immunohistochemistry (IHC) and *in situ* hybridization (ISH; DNA and/or RNA) staining methods to determine HPV status. Suspected HPV-associated tumors of the head and neck are traditionally stained using p16-IHC, which has a sensitivity of nearly 100% for transcriptionally active HPV, but lacks specificity, ranging from 79% to 95% (15). When p16-IHC is coupled with HPV DNA ISH and/or HPV E6/E7 RNA ISH, specificity increases to 90%–100% (15). For the purposes of the analytical performance validation of our ddPCR assay, comparator biopsy sample results were considered true positives or negatives based on concordant p16 and DNA and/or RNA ISH results (i.e., p16+/DNA ISH+, p16+/RNA ISH+ and p16+/DNA ISH+/RNA ISH+ = hrHPV true positive; p16-/DNA ISH-, p16-/RNA ISH-, and p16-/DNA ISH-/RNA ISH- = hrHPV true negative). Caveats for a few samples in which ISH staining results were not performed or unavailable are detailed in the results and discussion sections. When available, plasma HPV ctDNA testing results (*n* = 10) were compared between our ddPCR method and clinically available results from outside laboratories.

### Sample preparation and ctDNA isolation

Whole blood (10 mL) was collected in Cell-Free DNA BCT (Streck, La Vista, NE) tubes from healthy donors and double-centrifuged to produce PPP according to manufacturer’s protocol. Four milliliters of PPP was extracted on the EZ2 Connect (Qiagen, Hilden, Germany) according to manufacturer’s protocol using the EZ1&2 ccfDNA Kit (Qiagen) with an elution volume of 75 µL.

### Droplet generation and PCR amplification

Primer and probe sequences for HPV-16, -18, -31, -33, and -35 and the human gene ribonuclease P protein subunit p30 (RPP30) are summarized in Table 1. The PrimerQuest and OligoAnalyzer Tools (Integrated DNA Technologies [IDT], Coralville, IA) were utilized for novel primer and probe design for some genotypes. Considerations for selection and design of primer and probe sequences included gene target, GC content, avoidance of secondary structures, and amplicon size (i.e., 60-150 bp; ideally <100 bp). An additional consideration for downstream multiplexing necessitated selecting a single annealing temperature for workflow efficiency, which required scrutiny of concentrations of sodium, magnesium, enzyme, dNTP, and oligonucleotides. Screening of single-probe oligonucleotide sets, followed by pooled sets, resulted in the final selections detailed in Table 1. The ddPCR assay consists of two reactions (i.e., panels A and B) that were individually validated to support customized ordering options for clinical testing. In total, 18 primers and nine hydrolysis probes (IDT) were split across the two reactions/panels, primarily targeting HPV oncogenes (E6 and E7). The first reaction is referred to as “Panel A” and consists of eight primers and four probes detecting unique regions of the E2, E6, and E7 gene regions of HPV-16. A study by Bhambhani et al. (16) showed greater sensitivity and earlier cancer detection is possible with greater genome oligonucleotide coverage. With HPV-16 accounting for nearly 90% of cases of HPV(+)OPSCC (17), four primer sets were selected to ensure adequate gene coverage. The second reaction, referred to as “Panel B,” consists of 10 primers and five probes targeting the E6 and E7 oncogenes of four other hrHPV types. Two primer-probe sets were selected for HPV-18, one targeting the E6 gene and one targeting E7. The remaining three hrHPV genotypes (HPV-31, -33, and -35) have single primer and probe sets targeting E6 or E6/E7. An additional primer and probe set is included in each panel to detect a human housekeeping gene (RPP30), which serves as a positive extraction and amplification control for each sample, as well as an inhibition control.

**TABLE 1:** HPV ddPCR primer and probe sequences

Panel	HPV genotype	HPV gene target	NCBI reference genome	Oligo type	Sequence (5'→3')	Amplicon length (bp)	Source
A	16	E6	NC_001526.4	Forward primer	GGAACAACATTAGAACAGCA	90	(16)^*a*^
				Reverse primer	TTCTTCAGGACACAGTGG		
				Probe	56-FAM/ACAACAAAC/ZEN/CGTTGTGTGATTTG/3IABkFQ		
	16	E6/E7		Forward primer	AGAACACGTAGAGAAACC	95	(16)^*a*^
				Reverse primer	GAGATCAGTTGTCTCTGG		
				Probe	56-FAM/TATTCATGC/ZEN/AATGTAGGTGTATCTCC/3IABkFQ		
	16	E2		Forward primer	AACGAAGTATCCTCTCCTGAAATTATTAG	82	Novel design*^b^*
				Reverse primer	CCAAGGCGACGGCTTTG		
				Probe	5HEX/CACCCCGCC/ZEN/GCGACCCATA/3IABkFQ		
	16	E6		Forward primer	GAGAACTGCAATGTTTCAGGAC	81	Novel design*^b^*
				Reverse primer	TGTATAGTTGTTTGCAGCTCTGTG		
				Probe	5HEX/CAGGAGCGA/ZEN/CCCAGAAAGTTACCACAGTT/3IABkFQ		
A and B	N/A (human)	RPP30	NC_000010.11	Forward primer	AGATTTGGACCTGCGAGCG	65	(18)
				Reverse primer	GAGCGGCTGTCTCCACAAGT		
				Probe	5Cy5/TTCTGACCT/TAO/GAAGGCTCTGCGCG/3IAbRQSp		
B	18	E6	NC_001357.1	Forward primer	CACTATAGAGGCCAGTGC	65	(19)^*a*^
				Reverse primer	CTGCGTCGTTGGAGT		
				Probe	5HEX/CCTGTCGTG/ZEN/CTCGGTTGC/3IABkFQ		
	18	E7		Forward primer	GTGTGAAGCCAGAATTGAG	88	Novel design*^b^*
				Reverse primer	AAAGGACAGGGTGTTCAG		
				Probe	5HEX/AGTAGAAAG/ZEN/CTCAGCAGACGAC/3IABkFQ		
	31	E6	X05015.1	Forward primer	ACAACATAGGAGGAAGGTG	70	(19)^*a*^
				Reverse primer	ACTTGGGTTTCAGTACGAG		
				Probe	ROXN/TCCAACATGCTATGCAACGTCC/3IAbRQSp		
	33	E6/E7	J04353.1	Forward primer	CTGCACTGTGACGTGTA	88	Novel design*^b^*
				Reverse primer	GTCAGTTGGTTCAGGATATAAA		
				Probe	56-FAM/ATGAGAGGA/ZEN/CACAAGCCAACG/3IABkFQ		
	35	E6	M12732.1	Forward primer	GCTGAACGACCTTACAAAC	106	Novel design*^b^*
				Reverse primer	CACTCCGCTGTAATTCTTG		
				Probe	5ATTO590N/TGCTTTCTTCTACCTCGTTGCAC/3IAbRQSp		

^*a*^ Some sequence alterations were made to suit thermocycling conditions.

^*b*^ Oligo sets were designed in coordination with Integrated DNA Technologies (IDT) using the PrimerQuest Tool.

Following extraction, 8 µL of eluate was combined with 14 µL of ddPCR Multiplex Supermix-containing 1× master mix (Bio-Rad Laboratories, Hercules, CA), with final primer and probe concentrations of 900 nM and 250 nM, respectively, as well as dithiothreitol (DTT, Bio-Rad) at a final concentration of 4 mM. The plate was heat-sealed for 5 s at 180°C using a PX1 PCR Plate Sealer (Bio-Rad), vigorously vortexed and centrifuged for 10 s on a PerfectSpin P plate centrifuge (PeqLab VWR, Radnor, PA). Subsequently, the 96-well plate was placed onto an Automated Droplet Generator (AutoDG, Bio-Rad) for reaction partitioning into ~20,000 oil droplets. The partitioned reaction plate was heat-sealed as previously described, then placed on a VeritiPro Thermal Cycler instrument (Applied Biosystems, Waltham, MA) with thermocycling at the following parameters: 1 cycle at 95°C for 10 min; 45 cycles at 94°C for 30 s, 54°C for 90 s; 1 cycle at 98°C for 10 min; 1 cycle at 4°C for 15 min, followed by an indefinite hold at 4°C. Default ramp rates were used for all cycling steps, except for the annealing/extension step, which was slowed to a rate of 1.5°C/s.

### Droplet analysis and result interpretation

Following amplification, the 96-well plate was placed onto the QX600 droplet reader. Droplets were individually counted by passage in a single stream through a fluorometer. The QX Manager standard edition software (version 2.2.0.71, Bio-Rad) tallied the total number of droplets per reaction and the number of positive droplets for a given fluorophore. Data were fitted to a Poisson distribution to determine the absolute starting copy number in units of copies/μL in the reaction well. Each HPV genotype, as well as RPP30, was assigned a unique dye channel within each panel according to the probe-specific fluorophore, which allowed for differentiation of positive and negative droplets, as well as genotyping of positive droplets.

Fluorescence threshold values were set for each dye channel used within each panel. Each reaction was reviewed visually for the two-dimensional droplet pattern as well as for two quality control (QC) metrics: minimum total droplet count (≥10,000) and minimum concentration of RPP30 (≥500 fragments/mL). With successful QC, PPP concentrations of HPV ctDNA were calculated using the software-provided reaction concentration and a dilution factor accounting for the total volume of sample extracted, total elution volume, and eluate volume used with respect to the final 20-µL reaction volume. The concentration of HPV ctDNA was determined as fragments/mL of PPP, and these values were documented for each genotype detected by each panel. For HPV-16, the concentrations detected across both fluorescent dye channels (i.e., FAM and HEX) were combined to determine the total HPV-16 concentration detected by Panel A. Figure S1 shows the 2D droplet count and pattern of two representative HPV-16-positive samples.

### Assay validation

To validate the performance of the HPV ctDNA ddPCR assay, the following studies were completed: (i) precision (intra- and inter-assay), (ii) accuracy, (iii) analytical sensitivity (i.e., limit of detection [LoD]), (iv) reference range (i.e., normal value), (v) analytical measuring interval, and (vi) analytical specificity. All validation experiments were performed by testing samples using the workflow summarized above, from nucleic acid extraction to analysis and interpretation.

#### Precision

Analyte-negative PPP samples were spiked at three normalized concentrations (i.e., pooled, standardized concentrations) using synthetic gene fragments (gBlocks, IDT) from the four amplicon regions of HPV-16 representing Panel A and the two amplicon regions of HPV-18 representing Panel B. Samples were tested in triplicate on a single run (intra-assay) and in singlet on three separate days (inter-assay). Inter-assay precision experiments included operator and equipment variability, as available.

#### Accuracy

A limited number of clinical samples (*n* = 32) were available for accuracy testing, and contrived (i.e., spiked) samples (*n* = 48) were included to contribute to the data set. All samples described below were tested in singlet in a blinded manner. Analyte-negative PPP samples (*n* = 26) were spiked with one or more of the five HPV genotypes representing Panels A and B using gBlocks at concentrations spanning the analytical measuring interval. Sixty-nine percent (18/26) of samples were spiked between 10 and 100 fragments/mL, representing low positive samples. Unspiked samples (*n* = 22) were also tested by both Panels A and B. Clinical PPP samples (*n* = 32) were stored at <−70°C for up to 5 years from collection with a single freeze-thaw cycle prior to testing by Panels A and B. Clinical sample results were qualitatively compared (i.e., hrHPV positive or hrHPV negative) to IHC/ISH biopsy stain results. When available, results (*n* = 10) were compared with clinically available plasma ddPCR genotyping at an outside laboratory. Similarly, available corresponding formalin-fixed paraffin-embedded (FFPE) biopsy samples (*n* = 4) were processed for DNA from the blocks and tested by the in-house HPV ddPCR assay to correlate plasma genotyping results.

#### Analytical sensitivity

The limits of blank (LoB) were determined by testing two pools of analyte-negative PPP in four replicates over 3 days by both Panels A and B. Results from 24 data points for each target were subjected to the following equation for LoB determination: LoB = mean_blank_ + 1.645(SD_blank_) (20). To establish the limits of detection (LoDs), pools of analyte-negative PPP were spiked with normalized concentrations using gBlocks at 100 fragments/mL. A twofold dilution series was performed, and each level was extracted in triplicate and tested in duplicate by its corresponding panel (i.e., Panel A or Panel B) for a total of six replicates per genotype target per concentration. The LoD was established as the lowest concentration (i.e., highest dilution), where all six replicates were detected and quantified above the LoB for each target within both Panels A and B. The LoD was confirmed by spiking an additional 20 unique PPP samples at the previously established lowest concentration for each genotype. Data were then subjected to the following equation for final LoD determination: LoD = LoB + 1.645(SD_low concentration sample_) (20). Additionally, the lower limits of quantitation (LLoQ) were confirmed by assessing the lowest concentration in which the coefficient of variation (CV) was ≤25% across 20 unique replicates for each genotype.

#### Reference range

PPP samples (*n* = 20) from normal donors were tested in singlet by the ddPCR method described above for both Panels A and B.

#### Analytical measuring interval

To determine the quantitative range of the assay, pools of analyte-negative PPP were spiked with normalized concentrations using gBlocks at seven concentrations from 1 to 400,000 fragments/mL. Each level was extracted and tested in duplicate by Panels A or B for a total of four replicates per genotype target per concentration. Observed and expected quantitation results were compared with linear regression analysis and difference plots for correlation of results for each HPV genotype.

#### Analytical specificity

Testing of available HPV genotypes was performed to ensure specificity of primers and probes. Commercially available whole organism was extracted and sheared at 150 and 300 bp using a LE220-plus Focused-ultrasonicator system (Covaris, Woburn, MA) for the HPV following genotypes: 6b, 11, 16, 18, 31, 33, 39, 45, 51, 52, 66, and 68. Additional specificity testing was performed using a panel of 30 sheared nucleic acid extracts from various bacterial, fungal, parasitic, and viral organisms commonly found in PPP (Table S1). Inclusivity *in silico* analysis was performed using the NCBI GenBank database. Homology of Panels A and B primers and probes was determined using representative strains from sublineages for all genotypes as described by Burk et al., Galati et al., and Li et al. (21–23), with 342 different combinations of sequences analyzed.

## RESULTS

### Precision

Both intra- and inter-assay reproducibility of the HPV ctDNA ddPCR assay were evaluated by spiking analyte-negative PPP with gBlocks of HPV-16 and −18 amplicon regions. All observed results demonstrated 100% qualitative agreement with the expected result, and quantitative values demonstrated a standard deviation (SD) of 0.00–0.07 log_10_ fragments/mL across all genotype replicates.

### Accuracy

To assess the accuracy of the novel ddPCR assay, we first compared the results of clinical PPP samples to corresponding IHC/ISH biopsy results. Of the 32 clinical PPP samples tested, we observed a 90.6% (29/32) qualitative agreement with IHC/ISH stain results, with an 88.5% (23/26) positive percent agreement (PPA) and 100% (6/6) negative percent agreement (NPA) (Table 2). Additionally, 10 clinically available HPV ctDNA PPP test results reported in the electronic health record tested by an outside ddPCR assay were compared with our ddPCR assay. This yielded an overall agreement of 90% (9/10) (Table 3). Finally, results of four PPP samples were compared with corresponding FFPE biopsy samples, which were also tested by the in-house ddPCR assay, and this yielded an overall agreement of 75% (3/4) (Table 4).

**TABLE 2:** Comparison of plasma ddPCR to IHC/ISH biopsy stain results

	HPV genotype	Biopsy by IHC/ISH staining			
		p16+*^a^*	p16+/ISH+*^b^*	p16-*^a^*	p16-/ISH-*^b^*
**Plasma by in-house ddPCR** ^*f*^	HPV-16	2	20	0	0
	HPV-35	0	1	0	0
	Negative	0	3 *^c^^,d,e^*	4	2

^*a*^ ISH not available.

^*b*^ ISH testing varied among samples (i.e., DNA and/or E6/E7 RNA). These ISH assays detect multiple hrHPV genotypes (e.g., 16, 18, 31, 33, 35, 45, 51, 52, 56, 58, 59, 68, and 70) and do not offer genotypic differentiation.

^*c*^ All three samples were negative by both Panels A and B. Remaining plasma sample volume was insufficient for repeat testing by the in-house ddPCR assay.

^*d*^ One sample was also negative by the reference ddPCR assay in plasma.

^*e*^ One sample yielded an HPV-16 concentration of 12.88 frag/mL by the in-house ddPCR assay, which is >LoB but <LoD. A corresponding biopsy sample from the same patient was also tested by the in-house ddPCR, which was positive for HPV-16 at 201,993.14 frag/mL.

^*f*^ HPV genotypes 18, 31, and 33 were undetected by the in-house ddPCR assay.

**TABLE 3:** Comparison of in-house plasma ddPCR assay to clinically available HPV ctDNA ddPCR results^*a*^

	HPV genotype	Plasma by outside reference ddPCR	
		HPV-16	Negative
**Plasma by in-house ddPCR**	HPV-16	8	1^*b*^
	Negative	0	1

^*a*^ HPV genotypes 18, 31, 33, and 35 were undetected by both assays.

^*b*^ One sample was positive by the in-house ddPCR assay for HPV-16 at 31.14 frag/mL. Remaining plasma sample volume was insufficient for repeat testing. Sample was noted to have a corresponding biopsy that was p16+/RNA ISH+.

**TABLE 4:** Comparison of plasma ddPCR to FFPE biopsy ddPCR

	HPV genotype	FFPE biopsy by in-house ddPCR		
		HPV-16	HPV-35	Negative
**Plasma by in-house ddPCR** ^*a*^	HPV-16	2	0	0
	HPV-35	0	1	0
	Negative	1^*b*^	0	0

^*a*^ HPV genotypes 18, 31, and 33 were undetected in both sample types by the in-house ddPCR assay.

^*b*^ One PPP sample yielded an HPV-16 concentration of 12.88 frag/mL by the in-house ddPCR assay, which is >LoB but <LoD. The corresponding biopsy sample was positive for HPV-16 at 201,993.14 frag/mL. Remaining plasma sample volume was insufficient for repeat testing.

To further study the accuracy of the novel HPV ctDNA ddPCR assay, gBlocks for each HPV genotype detected by Panels A and B were spiked into pools of analyte-negative PPP. The assay correctly detected the expected genotypes in 100% (26/26) of samples and did not detect any HPV genotype in unspiked samples (22/22) for an overall agreement of 100% (48/48). Additionally, regression analysis plots for each HPV target yielded coefficient of determination (R^2^) values ≥ 0.95.

### Limits of blank and limits of detection

The limits of blank (LoBs) for each HPV genotype detected by Panels A and B were established by testing replicates of pools of analyte-negative PPP over 3 days. The resultant LoB values ranged from 0 to 1.38 droplets, which equaled 0.00–4.82 fragments/mL of PPP.

The limits of detection (LoDs) were initially established by testing six replicates of a six-level dilution panel of contrived (i.e., spiked) PPP samples representing each HPV genotype detected by Panels A and B. The highest dilution (i.e., lowest concentration) determined to be positive above the LoB in all six replicates was established as the initial LoD. Confirmation of the LoD was performed in 20 unique contrived PPP samples. Confirmation testing was performed to challenge assay robustness and highlight the variability of unique plasma samples that will be encountered during future routine clinical testing. The SD of values from the 20 low-concentration samples was used for final LoD determination in combination with the following equation: LoD = LoB + 1.645(SD_low concentration sample_). Final LoD values in fragments of HPV per milliliter of PPP were as follows: HPV-16, 17.54; HPV-18, 19.45; HPV-31, 17.83; HPV-33, 9.20; HPV-35, 7.71.

### Lower limits of quantitation

The lower limits of quantitation (LLoQs) were also confirmed by testing 20 unique PPP samples spiked with gBlocks for each HPV genotype at 100 fragments/mL (2.00 log_10_ fragments/mL). Percent CV values ranged from 17.58 to 23.52 across all five genotypes.

### Analytical measuring interval

**Fig 1:**
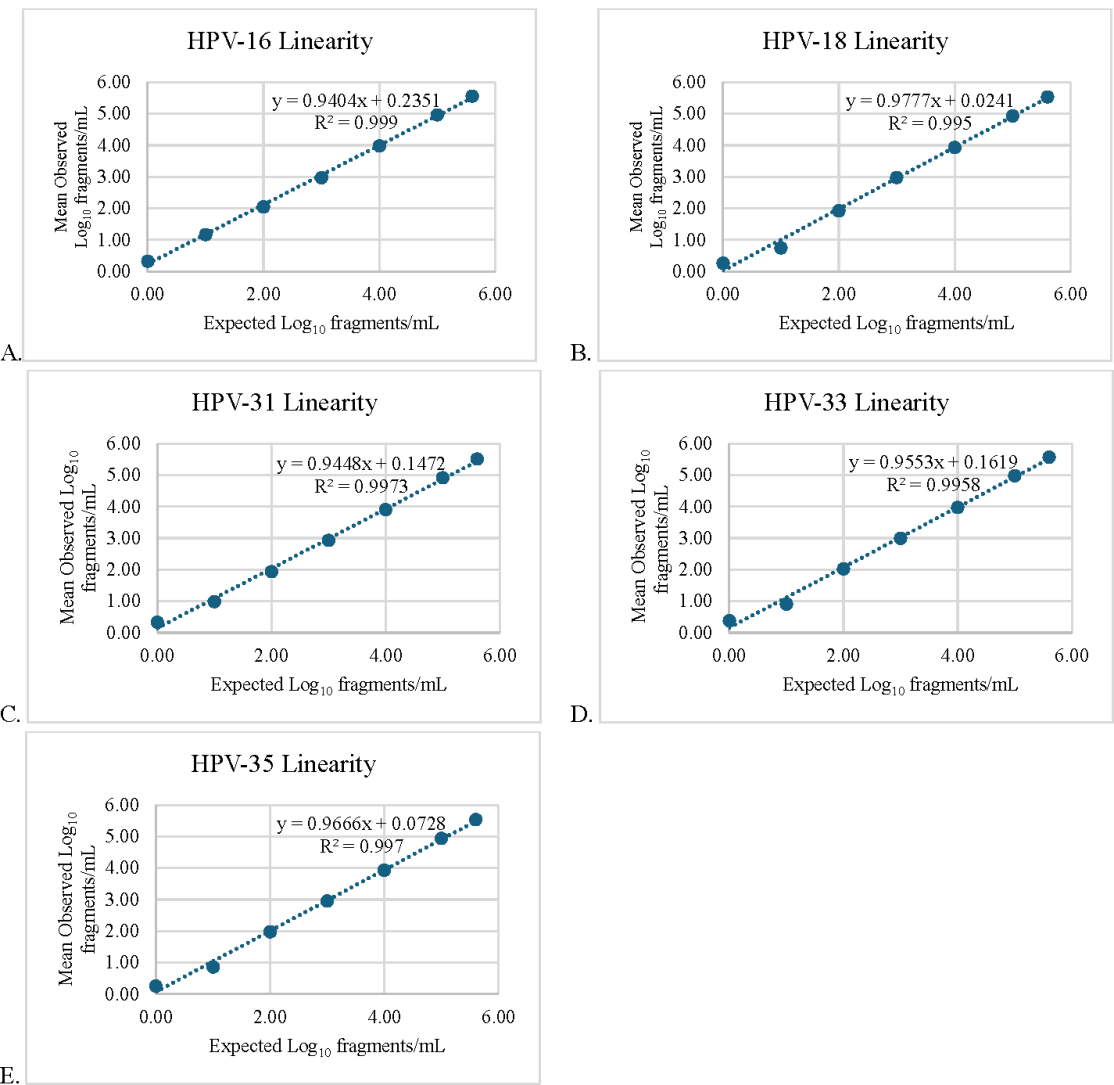
HPV ddPCR linearity plots for HPV-16 (A), HPV-18 (B), HPV-31 (C), HPV-33 (D), and HPV-35 (E). Analyte-negative plasma samples were spiked with target-specific synthetic gene fragments at known concentrations (range: 1–400,000 fragments/mL) and tested by the in-house HPV ddPCR assay. The average of four replicates was plotted for linearity with resultant R2 values ≥0.99 for each HPV genotype. y, slope; R², coefficient of determination.

Determination of the analytical measuring interval (i.e., linearity) was performed by testing seven levels of analyte-negative PPP pools spiked with gBlocks for each HPV genotype from 1 to 400,000 fragments/mL. Each level was extracted and tested in duplicate, resulting in four replicates per level. Regression analysis plots (Fig. 1) of the average concentration per level generated R^2^ values ≥ 0.99 and slope values of 0.94–0.98 throughout the entire range (0.00–5.60 log_10_ fragments/mL) for each HPV genotype. Although linearity was achieved throughout the entire range of contrived samples, lower and upper limits of quantitation were finalized as 100 fragments/mL (2.00 log_10_ fragments/mL) and 309,600 fragments/mL (5.49 log_10_ fragments/mL), respectively, based on LLoQ experiments in unique samples and the manufacturer’s recommended dynamic range (24) of the QX600 instrument.

### Analytical specificity and reference range

Assay specificity was assessed by testing nucleic acid extracted from a robust panel of 30 bacterial, fungal, parasitic, and viral organisms commonly found in plasma, as well as 8 HPV genotypes not expected to be detected by Panels A nor B. No cross-reactivity was detected above the established LoD for any target. Assay-specific HPV genotypes available from commercial vendors (e.g., HPV-16,-18, -31, and -33) were all correctly detected and genotyped as expected.

An *in silico* analysis was performed using the NCBI GenBank database. Homology of Panels A and B primers and probes was assessed using 22 different HPV-16 strains representing all four lineages (A–D), nine strains of HPV-18 representing all three lineages (A–C), seven strains of HPV-31 representing all three lineages (A–C), five strains of HPV-33 representing all three lineages (A–C), and four strains of HPV-35 representing the single lineage (A). In total, 342 different combinations of primers, probes, and target sequences were analyzed. Homology of primers and probes ranged from 89.5% to 100% across all HPV-16 sublineages. Of the four primer/probe sets targeting different regions of the HPV-16 genome, at least one set yielded 100% homology across all 22 HPV-16 strains assessed. Notably, HPV-16 lineage A, which is the most common lineage detected in HPV-16 positive head and neck cancers (22), showed 94.7 – 100% homology across strains analyzed. For Panel B, homology of primers and probes ranged from 88.9 to 100% for HPV-18. At least one of the two HPV-18 primer/probe sets was 100% homologous to all representative sublineage strains analyzed (*n* = 9). Primer and probe homology for the remaining Panel B genotypes (e.g., HPV-31, -33, and -35) ranged from 94.7% to 100% across all strains analyzed (*n* = 16). All 20 healthy donor samples tested as part of the reference range experiment were negative by both Panels A and B.

## DISCUSSION

The growing number of HPV-associated cancers has necessitated access to testing options, especially for detecting and monitoring HPV(+)OPSCC. Importantly, this study demonstrated robust performance of an HPV ctDNA ddPCR assay, which performed well when compared with p16-IHC/ISH tumor biopsy results and clinically reported, commercially available HPV ctDNA PPP results. It also demonstrated strong agreement between PPP and tumor biopsy FFPE.

Our study showed a strong overall agreement (90.6%; 29/32) between p16-IHC biopsy results, with or without HPV-ISH staining, and the HPV ctDNA ddPCR assay developed in-house. The three discrepant samples were all p16-IHC and HPV-ISH positive with corresponding plasma samples that were negative by our ddPCR assay. None of the three discrepant samples had sufficient remaining plasma volume to repeat ddPCR testing. One discrepant sample (p16+/DNA ISH+/RNA ISH+) yielded a ddPCR plasma concentration of 12.88 fragments/mL of HPV-16 ctDNA, which is a value above the LoB but below the LoD for HPV-16. The corresponding FFPE biopsy sample was available for testing by the ddPCR assay and yielded a positive result for HPV-16 at 201,993.14 fragments/mL. This suggests the plasma was likely positive for HPV-16 ctDNA at a concentration below the 95% LoD for the assay. The second discrepant sample (p16+/RNA ISH+, in-house ddPCR negative) was also negative for HPV-16,-18, -31, -33, and -35 by routine clinical HPV ctDNA ddPCR testing at an outside reference laboratory. The third discrepant sample (p16+/DNA ISH+/RNA ISH+) did not have comparative routine clinical HPV ctDNA ddPCR testing at an outside reference laboratory, nor was a corresponding biopsy sample available for testing by our ddPCR assay. These ISH assays detect additional HPV genotypes that are not covered by our ddPCR assay and do not differentiate which HPV genotype is detected (15). These discrepant samples may indicate (i) low or undetectable concentrations of circulating HPV ctDNA in PPP, (ii) an OPSCC tumor not caused by one of the hrHPV genotypes detected by our in-house ddPCR or the reference ddPCR assay, (iii) an OPSCC tumor not caused by HPV, or (iv) a false-negative.

As compared with commercially available, clinically reported HPV ctDNA testing results, our ddPCR assay showed an overall agreement of 90% (9/10). The single discrepant sample was negative by the reference ddPCR assay but positive for HPV-16 by our test at a concentration of 31.14 fragments/mL. Interestingly, the corresponding tissue biopsy was p16+/RNA ISH+. Remaining PPP sample volume was insufficient for repeat testing. The tissue biopsy was unavailable for testing by our ddPCR assay, but future testing and algorithm development to include biopsy testing may assist when the results of plasma HPV ctDNA and biopsy are discordant.

Finally, testing of PPP and corresponding FFPE tumor biopsy tissue by our novel ddPCR assay demonstrated an overall agreement of 75% (3/4). The single discrepant sample was HPV-16 positive in the biopsy sample but yielded an HPV-16 concentration above the LoB but below the LoD in plasma. The tumor burden (i.e., the volume of overall tumor within the patient at the time of testing, inclusive of the primary tumor, as well as any nodal or distant metastases) was not available for this sample, but a strong correlation between tumor burden and plasma HPV ctDNA concentrations has been described (25). It is possible that this patient had a low tumor burden resulting in a low HPV ctDNA PPP concentration.

An HPV ctDNA ddPCR assay subjected to a robust analytical performance validation has clinical utility not only for disease monitoring as stated but also for initial diagnosis (26) and HPV genotyping. Novel HPV-targeted therapies, such as therapeutic vaccines, may be HPV-16 specific, creating a need for specificity in understanding the HPV genotype associated with an individual cancer. While this study focused on validation of PPP, the ability to perform testing on tissue would offer critical information currently not available with ISH, for which most clinical testing consists of “cocktails” that provide hrHPV positive or negative results without genotypic differentiation. Areas of future study include validation of biopsy, including fine needle aspiration and core, as well as surgical specimens in HPV(+)OPSCC and other HPV-associated malignancies, such as anal and cervical carcinoma.

This study has several limitations. First, we compared PPP HPV ctDNA results to the most widely used testing methods for HPV(+)OPSCC (i.e., p16-IHC and HPV-ISH) with a small clinical data set (*n* = 32). Among the clinical samples, none were positive for HPV-18, -31, or -33, thereby limiting the conclusions that can be made on the test’s performance for these genotypes. However, we supplemented accuracy data by testing contrived samples (*n* = 48) and observed 100% concordance with the expected results, including 16 samples that were spiked with HPV-18, -31, -33, and -35. An additional six samples were included as part of analytical specificity (Table S1) with 100% concordance with the expected results. Second, co-testing all samples by both the in-house ddPCR and outside, reference ddPCR assays was not feasible due to limited sample volume. However, the small sample set (*n* = 10) demonstrated 90% agreement between the two assays. Finally, correlation with clinical outcomes was not available.

Development and validation of this highly complex assay included several challenges and considerations for future development efforts. While genotype selection was driven by current data on the primary genotypes associated with HPV(+)OPSCC tumors (7–14), we recognize that at least nine other hrHPV genotypes have oncogenic potential. The assay was designed to maximize sensitivity and specificity for the genotypes associated with >95% of HPV(+) OPSCC tumors. Additional panels could be added in the future to cover other genotypes, in the event that epidemiologic evidence suggests they are increasing in prevalence.

Data summarized in this report detail the analytical performance validation of a novel ddPCR assay developed for the detection and quantitation of ctDNA from 5 hrHPV genotypes in PPP. Future studies to assess assay utilization in the context of different clinical presentations and specimen types may provide insight for diagnosis, management, and surveillance of HPV(+)OPSCC patients.

## SUPPLEMENTAL MATERIAL

Supplemental table and figure jcm.00585-25-s0001.docx Table S1 and Fig. S1.

ASM does not own the copyrights to Supplemental Material that may be linked to, or accessed through, an article. The authors have granted ASM a non-exclusive, world-wide license to publish the Supplemental Material files. Please contact the corresponding author directly for reuse.
