# Supplementary material for: Detection, quantitation, and genotyping of human papillomavirus circulating tumor DNA by droplet digital PCR
Source: J Clin Microbiol. 2025 Aug 19;63(9):e00585-25. doi: 10.1128/jcm.00585-25 (PMC12421868; doi:10.1128/jcm.00585-25)
Supplement: Supplemental table and figure — Table S1 and Fig. S1. [file jcm.00585-25-s0001.docx]

Supplemental Figure 1. Representative 2D Droplet Plots for Samples Positive for HPV-16

| A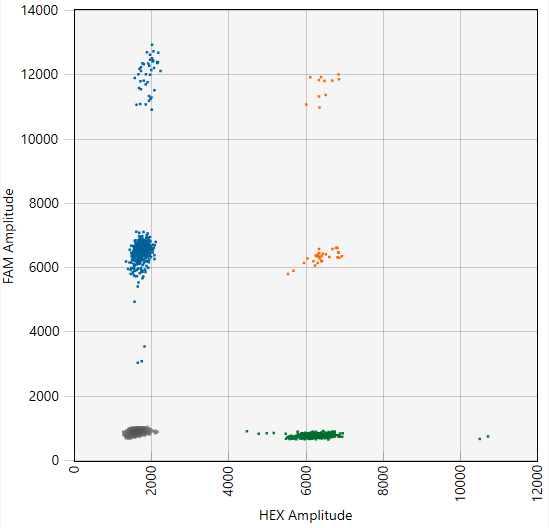 | B 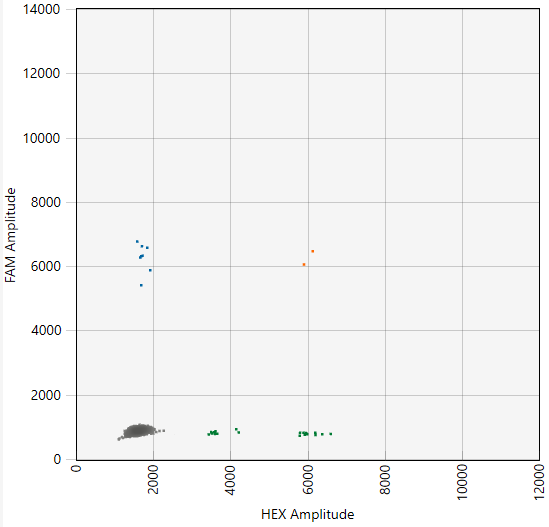 |
| --- | --- |

Supplementary Figure 1: These 2D plots (A, B) represent droplets that contain FAM-labeled amplicon (y-axis; blue dots), HEX-labeled amplicon (x-axis; green dots), droplets with one or more FAM- and HEX-labeled amplicon (orange dots), as well as droplets without either fluorophore (i.e., negative; gray dots). Plot A shows a strong HPV-16 positive sample, evidenced by 529 FAM-droplets (blue and orange dots) and 594 HEX-droplets (green and orange dots), representing 1,639.97 and 1,844.37 HPV-16 fragments/mL, respectively, for a total of 3,484.34 HPV-16 fragments/mL of plasma. Plot B shows a weaker HPV-16 positive sample, evidenced by 10 FAM-droplets (blue and orange dots) and 22 HEX-droplets (green and orange dots), representing 31.36 and 69.02 HPV-16 fragments/mL, respectively, for a total of 100.38 HPV-16 fragments/mL of plasma.

Supplemental Table 1. Analytical Specificity: Wet-Lab Testing by HPV ddPCR

| Identification *^a^* | Target | Concentration (genomes/µL) | Panel A HPV Result | Panel B HPV Result |
| --- | --- | --- | --- | --- |
| UM-SCC-104, SCC072 | HPV-16 | 0.1ng/µL | HPV-16 Positive | Negative |
| Bio-Rad HPV16C | HPV-16 | 2.5E+04 | HPV-16 Positive | Negative |
| ATCC VR-3240SD | HPV-16 | 28.5 | HPV-16 Positive | Negative |
| UM-SCC-105, SCC189 | HPV-18 | 0.1ng/µL | Negative | HPV-18 Positive |
| Bio-Rad HPV18C | HPV-18 | 2.5E+04 | Negative | HPV-18 Positive |
| ATCC VR-3241SD | HPV-18 | 26.0 | Negative | HPV-18 Positive |
| ATCC VR-3256SD | HPV-31 | 22.5 | Negative | HPV-31 Positive |
| Seegene VP-62-31 | HPV-31 | 5.00E+07 | Negative | HPV-31 Positive |
| Seegene VP-62-33 | HPV-33 | 5.00E+07 | Negative | HPV-33 Positive |
| Millipore, G4031 | Human gDNA | 1.14E+02 | Negative | Negative |
| Seegene VP-62-39 | HPV-39 | 5.00E+07 | Negative | Negative |
| Seegene VP-62-45 | HPV-45 | 5.00E+07 | Negative | Negative |
| Seegene VP-62-51 | HPV-51 | 5.00E+07 | Negative | Negative |
| Seegene VP-62-52 | HPV-52 | 5.00E+07 | Negative | Negative |
| Seegene VP-62-66 | HPV-66 | 5.00E+07 | Negative | Negative |
| Bio-Rad HPV68C | HPV-68 | 1.25E+07 | Negative | Negative |
| ATCC 45150D | HPV-6b | 2.78E+07 | Negative | Negative |
| ATCC 45151D | HPV-11 | 3.88E+07 | Negative | Negative |
| ATCC 43069 | *Neisseria gonorrhea* | 7.00E+05 | Negative | Negative |
| Clinical isolate | *Candida auris* | 1.15E+04 | Negative | Negative |
| ATCC 1022 | *Aspergillus fumigatus* | 1.00E+04 | Negative | Negative |
| ATCC 50174D | *Toxoplasma gondii* | 4.35E+03 | Negative | Negative |
| ATCC VR-538DQ | CMV | 3.65E+04 | Negative | Negative |
| CAP 4-A-13-1-19, CRP-1 | *Acinetobacter baumannii* | 1.47E+05 | Negative | Negative |
| ATCC 25285, CRP-3 | *Bacteroides fragilis* | 1.68E+05 | Negative | Negative |
| ATCC 35210, CRP-4 | *Borrelia burgdorferi* | 2.28E+04 | Negative | Negative |
| CAP 4-A-15-1-2, CRP-8 | *Enterococcus faecium* | 5.16E+04 | Negative | Negative |
| ATCC 25922, CRP-9 | *Escherichia coli* | 7.04E+04 | Negative | Negative |
| ATCC 9006, CRP-11 | *Haemophilus influenzae* | 2.76E+05 | Negative | Negative |
| CAP 4-A-14-B-8, CRP-13 | *Klebsiella oxytoca* | 1.00E+05 | Negative | Negative |
| ATCC 700603, CRP-14 | *Klebsiella pneumoniae* | 2.32E+04 | Negative | Negative |
| ATCC 27583, CRP-22 | *Pseudomonas aeruginosa* | 7.16E+04 | Negative | Negative |
| ATCC 6919, CRP-41 | *Cutibacterium acnes* | 9.64E+05 | Negative | Negative |
| ATCC 14990, CRP-42 | *Staphylococcus epidermidis* | 2.67E+05 | Negative | Negative |
| BAA-977, CRP-49 | *Staphylococcus aureus* | 2.35E+05 | Negative | Negative |
| ATCC 33152, CRP-137 | *Legionella pneumophila* serogroup 1 | 6.55E+04 | Negative | Negative |
| ATCC VR-847D, CRP-83 | Human adenovirus 3 | 4.00E+06 | Negative | Negative |
| ABI 08-923-000, CRP-D5 | Human Herpesvirus-6 (Strain Z29) | 1.00E+03 | Negative | Negative |
| ABI 08-926-000, CRP-F6 | EBV (Strain B95-8) | 1.00E+03 | Negative | Negative |
| ATCC VR-735, CRP-132 | Herpes simplex virus Type 1 | 1.97E+07 | Negative | Negative |
| ATCC VR-540, CRP-133 | Herpes simplex virus Type 2 | 1.55E+07 | Negative | Negative |
| ATCC 3245D, CRP-135 | HIV - human immunodeficiency virus | 5.00E+08 | Negative | Negative |
| ATCC VR-3232SD, CRP-136 | Hepatitis B virus (HBV) | 9.14E+07 | Negative | Negative |
| ATCC VR-3235SD, CRP-137 | Hepatitis C virus (HCV) | 1.15E+08 | Negative | Negative |
| ABI 08-765000, CRP-142 | Human Herpesvirus-7 | 1.00E+04 | Negative | Negative |
| Zeptometrix, CRP-45 | Parvovirus B19 | 1.85E+07 | Negative | Negative |
| ATCC 3257SD, CRP-144 | Hepatitis A virus (HAV) | 3.80E+04 | Negative | Negative |

*^a^* CRP: Cross-reactivity panel; internal set of nucleic acids used for assay development and validation at our institution.
